# Chloroform

**Published:** 1858-12

**Authors:** 


### CHLOROFORM.

When equal volumes of chloroform and colorless concentrated sulphuric acid (the strong commercial oil of vitriol answers very well) are shaken together in a glass stoppered vial there should be no color imparted to either liquid, or but a faint tinge of color, after twelve hours standing together. Nor should there be any heat developed in the mixture at the time of shaking it first. Any particles of dust, or cork, or other organic matter that may have been in the vial used, or in the chloroform, will, by reaction of the acid, produce a tinge of color in the acid or its separation from the chloroform, corresponding to the quantity of such particles present, and therefore in the application of the test care must be taken to avoid any such particles. Or if the acid be only faintly tinged at the end of twelve hours' contact with chloroform, it may be attributed to some collateral accidental cause. But if at the end of twelve hours, or sooner, the acid becomes yellow, or brown, or any dark color, it should be unhesitatingly rejected. If the mixture of acid and chloroform should become warm on shaking first, an admixture of alcohol would be indicated. One or two fluid drachms of chloroform spontaneously evaporated from a clean surface of glass or porcelain, or from a piece of clean unsized paper, should leave no odor after it. Commercial chloroform generally will turn the acid brown within two or three hours, and will often render it black and tarry-looking within two or three days; whilst with chemically pure chloroform there is absolutely no reaction within many days.

